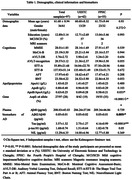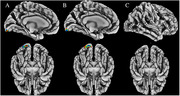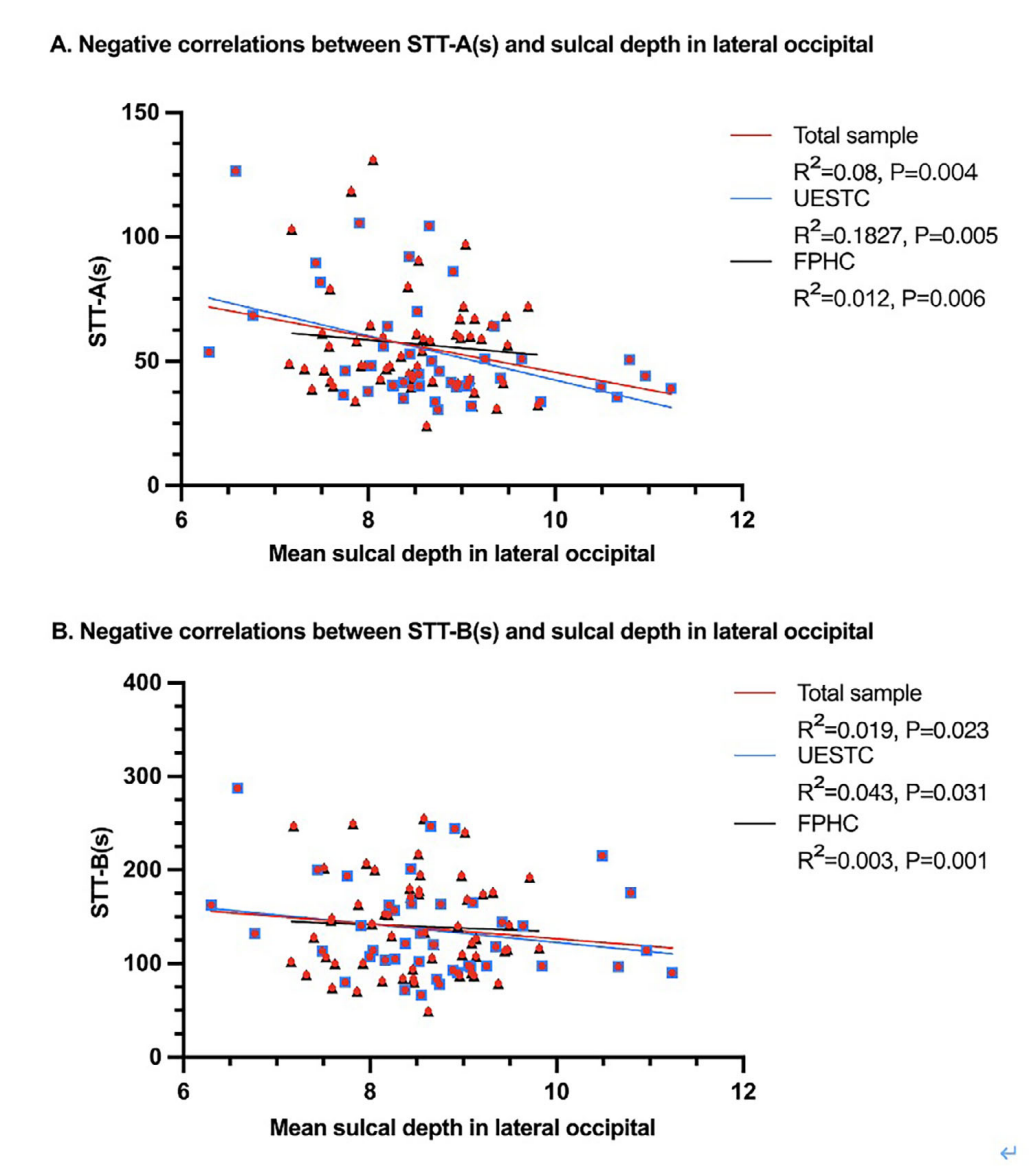# Correlation analysis of ApoB, ApoA1 and ApoB/ApoA1 with cortical morphology in patients with memory complaints

**DOI:** 10.1002/alz.087807

**Published:** 2025-01-09

**Authors:** Jiayu Wang, Lei Chen, Ziqi Wang, Mingjun Duan

**Affiliations:** ^1^ Zunyi Medical University, zunyi, Guizhou China; ^2^ Zunyi Medical University, Zunyi, Guizhou China; ^3^ The Clinical Hospital of Chengdu Brain Science Institute, MOE Key Lab for Neuroinformation, School of Life Science and Technology, University of Electronic Science and Technology of China, Chengdu, Sichuan China; ^4^ The Fourth People’s Hospital of Chengdu, Chengdu, Sichuan China

## Abstract

**Background:**

Apolipoproteins and cortical morphology are closely associated with memory complaints, and both may contribute to the development of Alzheimer’s disease.

**Method:**

A total of 97 patients from the University of Electronic Science and Technology (UESTC) (n=42) and the Fourth People's Hospital of Chengdu (FPHC) (n=55) were grouped based on recruitment location, and underwent neuropsychological tests. ApoB, ApoA1, ApoB/ApoA1, plasma Alzheimer’s biomarker, apolipoprotein E (ApoE) genotyping, 3T magnetic resonance imaging. The CAT12 toolbox (SPM12) was performed for the calculation of each patient's cortical morphology index based on structural MRI data, and the cortical morphology index and apolipoproteins were analyzed by multiple regression analysis.

**Result:**

Significant positive correlations were found between ApoB and sulcal depth in the lateral occipital cortex for the UESTC and total sample groups (p<0.0001). Sulcal depth in the lateral occipital cortex showed negative correlations from the Shape Trails Test Part A and B in the UESTC, the FPHC and total sample groups. In the FPHC group, the delayed recall of Auditory Verbal Learning Test, Animal Fluency Test and Boston Naming Test were positively correlated with the sulcal depth.

**Conclusion:**

ApoB is associated with the sulcal depth in the lateral occipital cortex, potentially relating to speed/executive function in individuals with memory complaints.